# Donald P. Kent Award, Robert W. Kleemeier Award, and James Jackson Outstanding Mentorship Award Lectures

**DOI:** 10.1093/geroni/igaf122.1249

**Published:** 2025-12-31

**Authors:** Marilyn Gugliucci

## Abstract

The Donald P. Kent Award lecture will feature an address by the 2024 Kent Award recipient Peter Lichtenberg, PhD, FSGA of the Wayne State University. The Robert W. Kleemeier Award lecture will feature an address by the 2024 Kleemeier Award recipient Jiska Cohen-Mansfield, PhD, FGSA of Tel Aviv University. The James Jackson Outstanding Mentorship Award lecture will features an address by the 2024 Jackson Award recipient Lisa Barnes, PhD, FGSA of Rush University. The Kent Award is given annually to a member of the Gerontological Society of America who best exemplifies the highest standards of professional leadership in gerontology through teaching, service, and interpretation of gerontology to the larger society. The Kleemeier Award is given annually to a member of the Gerontological Society of America in recognition for outstanding research in the field of gerontology. The Jackson Award is given annually to a member of the Gerontological Society of America in recognition outstanding commitment and dedication to mentoring minority researchers in the field of aging.

